# The burden of entheseal involvement in systemic lupus erythematosus: a comparative ultrasonograghic study

**DOI:** 10.1007/s10067-023-06675-9

**Published:** 2023-08-14

**Authors:** Ahmed Emerah, Shaimaa Mostafa, Lobna Kotb, Yomna Amer, Basma Ismail, Shymaa A. Sarhan

**Affiliations:** Rheumatology and Rehabilitation Department, Faculty of Medicine, Zagazig University, Zagazig, Egypt

**Keywords:** Enthesitis, Psoriasis, Systemic lupus erythematosus, US

## Abstract

**Background:**

Imaging is crucial for identifying and diagnosis of the musculoskeletal (MSK) symptoms, which are one of the most typical manifestations of systemic lupus erythematosus (SLE). For the joints, tendons, and entheseal sites, ultrasonography has shown to be sensitive and accurate for the diagnosis of both inflammation and structural damage.

**Aim:**

The goal of the current investigation is to determine the prevalence and the distribution of entheseal abnormalities in SLE patients, using musculoskeletal ultrasonography (MSUS) and to assess the relationship between entheseal sonographic changes and the SLE disease activity.

**Patients and methods:**

One hundred sixty-eight subjects were studied (56 SLE patients, 56 psoriatic arthritis (PSA) patients, and 56 normal cases). To compare the frequency and the distribution of entheseal involvement, high-resolution MSUS was conducted to assess the entheseal sites of all patients in accordance with the Madrid Sonographic Enthesitis Index (MASEI).

**Results:**

Clinical enthesitis was detected in 39.3% of the SLE patients using the Leeds Enthesitis Index compared to 71% detected via US examination, indicating a high proportion of subclinical enthesitis in our SLE patients. The most frequently affected enthesis was the distal insertion of the patellar tendon at the tibial tuberosity which was detected in 41% of SLE patients. Enthesitis was significantly more frequent in PSA patients (100%) compared to SLE patients (71.4%) (*p* < 0.05) and more significantly frequent in SLE patients compared to the healthy controls (19.6%). There was a significant correlation between MASI and SLEDAI scores (*r* = 0.250*, *p* = 0.048) and the total protein in 24 h (*r* = 0.289*, *p* = 0.031). In addition, there was an inverse significant correlation between MASEI and serum albumin (*r* =  − 0.324*, *p* = 0.015).

**Conclusion:**

In SLE patients, enthesitis is frequently clinical and ultrasound-verified. The most impacted enthesis is at the insertion of the quadriceps tendon. Enthesitis presence and the rise in the MASI score can serve as indicators of the severity of the SLE disease.

**Table:** 

**Key Points** • *The most impacted entheseal site lies at the insertion of the quadriceps tendon.* • *The presence and the rise in MASEI score can serve as indicators of the severity of the SLE disease.*

## Introduction

Systemic lupus erythematosus is a chronic multisystem autoimmune disease characterized by the production of a wide range of autoantibodies against nuclear, cytoplasmic, and cell surface antigens resulting in tissue damage and a variety of clinical manifestations [1, 2]. More than 50% of patients initially appear with musculoskeletal symptoms, and up to 95% of patients experience them [3, 4].

Patients with SLE typically have non-deforming, non-erosive arthritis, with some also showing signs of synovitis and tenosynovitis [5]. Notable is the incidence of arthralgia in SLE patients without clinically discernible synovitis [6]. Joint pain in SLE patients significantly lowers the quality of life and causes function loss [7]. Recent studies using ultrasonography have shown that SLE may encompass non-synovial tissues including tendon or ligament insertion sites [8, 9].

Detecting enthesopathy may be challenging due to the low sensitivity and specificity of clinical diagnostics; however, ultrasound appears to be a viable method. For detecting structural changes to the enthesis such as erosion and calcification and inflammatory changes such as bursitis or increased tendon thickness, all can be highly assessed by the US grey scale [10–12].

Enthesitis is a main pathogenic process in spondyloarthritis and is not just a localized inflammation. According to reports, 35 to 50% of psoriatic arthritis patients experience enthesitis [13].

So, the current research was aimed at detecting the entheseal changes in patients with systemic lupus erythematosus in comparison with psoriatic arthritis using musculoskeletal ultrasound Furthermore, the current study was aimed at evaluating the association between subclinical entheseal sonographic changes and SLE disease activity.

## Patient and method

This cross-sectional study was conducted on 168 subjects divided into three groups (56 SLE patients, 56 PSA patients, and 56 normal subjects). The patients were selected from outpatient clinics of Rheumatology Department at Zagazig University Hospitals after fulfilling the ethical considerations. All patients were above 18 years and had stable therapy in the 4 weeks preceding the evaluation. All systemic lupus erythematosus patients were diagnosed according to the ACR/EULAR SLE criteria [14]. Patients who underwent previous surgery or procedural intervention at the sites of an ultrasound examination or had ankle or knee synovitis at the time of the clinical evaluation or engaged in strenuous physical activity in the 4 weeks before the evaluation were excluded from the study.

A thorough medical history was taken, followed by a general and local clinical examination. SLEDAI-2 K (the Systemic Lupus Erythematosus Disease Activity Index) was calculated [15]. Using the Leeds Enthesitis Index (LEI) score, which evaluates six sites bilaterally, enthesitis was clinically diagnosed. Greater entheseal affection is represented by a higher score [16]. Complete blood count (CBC), C reactive protein (CRP), erythrocyte sedimentation rate (ESR), liver function tests, kidney function tests, and estimation of 24-h urine proteins were among the routine laboratory tests. Serum complements (C3 and C4), anti-dsDNA, and anti-nuclear antibody (ANA) were all measured.

### Musculoskeletal ultrasound evaluation

The US examination uses a high-frequency linear probe (10–18 MHz) that operates at a 10-MHz Doppler frequency. The Madrid Sonographic Enthesitis Index, which investigates six enthesis locations bilaterally in each patient including the brachial triceps tendons, distal quadriceps, proximal and distal patellar ligaments, distal Achilles tendon, and proximal plantar fascia, has proposed definitions for enthesitis in spondyloarthritis [17]. The grey scale and the power Doppler (PD) were used to scan the entheses bilaterally in both longitudinal and transverse planes.

The essential enthesis lesions of each site, including their structure, thickness, calcifications, erosions, and bursae, were assessed using ultrasound imaging, and power Doppler signals were also used for assessing enthesis and bursa.

The maximal anteroposterior diameter in millimeters as determined on the axial scan close to the bone insertion is referred to as thickness. The loss of the fibrillar pattern, a hypoechoic appearance, or the fusiform thickening of the enthesis was considered pathologic structural changes. Enthesophytes were characterized as a step-up bony protrusion at the end of the normal bone profile, while a cortical disruption with a step-down contour defect was used to define the bone erosion. Calcifications were measured and categorized according to size at the point where the enthesis was inserted. Power Doppler ultrasound was used to analyze the blood flow in each enthesis.

The MASEI score categorizes each of the calcifications: Doppler signal and bone erosion (0–3). As for the assigning tendon thickness, the tendon structure and bursa were either a 0 or a 1 (0 or 3). The total MASEI score was calculated using 12 entheses and had a maximum possible value of 136 [17].

### Patient positioning

For the evaluation of their triceps tendon enthuses, the individuals were seated in front of the examiner with their shoulders internally rotated and their elbows flexed 90°. During the evaluation of the knee entheses (insertion of the quadriceps tendon at the upper patella, proximal and distal insertion of the patellar tendon), patients had a neutral position, keeping a supine manner on the examination bed with the knee flexed at 30°. Patients were placed in a prone posture with their feet in a neutral position dangling over the side of the examination bed for the evaluation of the ankle entheses (Achilles tendon insertion at the calcaneus and the plantar fascia origin) [17].

### Statistical analysis

Using IBM Corp., version 23.0, all data were gathered, tabulated, and statistically analyzed. The mean, standard deviation, or median (range) was used to convey quantitative data and the number and percentage of qualitative data. To make a comparison between more than two sets of normally distributed variables, the *F* and *T* tests were employed. In order to compare two sets of non-normally distributed variables, the Mann–Whitney *U* test was used. When appropriate, the chi-square test or Fisher exact test was used to compare the percentage of categorical variables. A *p*-value < 0.05 was considered statistically significant.

## Results

The results of the study showed that there is no significant difference between SLE patients, PSA patients, and healthy controls regarding their age, sex distribution, or disease duration.

The clinical manifestations showed that about 85.7% of SLE patients had arthralgia, 78.6% had hair loss, 57.1% had photosensitivity, 17.9% had skin rash, 17.9% had ulcers, 10.7% had arthritis, 7.1% had fever, and 3.6% had psychosis. According to SLEDIA-2 K, 67.9% of SLE patients were mildly active, 21.4% were moderately active, while10.7% were inactive as indicated in Table 1.

**Table 1:** Demographic characteristics and clinical and laboratory data of studied SLE patients, PSA patients, and healthy controls

Variables	Studied groups		*t*/*u*	*p*-value
	SLE *n*. 56	PSA *n*. 56		
Age per years				
Mean ± SD	35.1 ± 8.8	38.9 ± 9	2.78	0.065
Range	20–50	21–50		
Sex				
Females	56 (100.0%)	50 (89.3%)	0.059	0.051
Males	0	6 (10.7%)		
Disease duration (years)				
Median (range)	75.5 (1–20)	5 (2–15)	0.79	0.43
White blood cells	6.3 (2.7–12)	6.6 (3.9–15.4)	0.501	.62
Hemoglobin	11.7 ± 1.4	12.02 ± 1.4	1.2	0.22
Total platelet count	282 ± 61	275 ± 60	0.61	0.54
Blood urea	12 (4.8–33)	11.4 (6.5–22)	1.62	0.105
Serum creatinine	0.7 (0.49–1.79)	0.7 (0.5–0.85)	0.32	0.75
Serum albumin	4.1 ± 0.45	4.3 ± 0.37	1.9	0.062
AST	16.2 (12–40)	18.3 (10.3–71.9)	1.78	0.075
ALT	15.2 (5.7–39.7)	18.3 (7.2–84.3)	1.88	0.057
LEI mean ± SD	0.94 ± 1.2	3.3 ± 1.7	2.23	0.0001
Median (range)	0 (0–4)	3.5 (0–6)		
Therapy	HDQ: *n*. 34 Azathioprine: *n*. 20 Cyclophosphamide: *n*. 10 Mycophenolate mofetil: *n*. 26 Corticosteroids: large dose (> 40 mg/day) *n*. 30; low dose: *n*. 20	NSAIDS: *n*. 41 MTX: *n*. 48 LEF: *n*. 12 Anti-IL 17: *n*. 28 JAK I: *n*. 6		

*t* = Student *t* test of significance, *u* = Mann–Whitney *u*, *p* > 0.05 not significant

*ALT* alanine aminotransferase, *AST* aspartate aminotransferase, *LEI* Leeds Enthesitis Index, *AZA* azathioprine, *CYC* cyclophosphamide, *MMF* mycophenolate mofetil, *CS* corticosteroids, *MTX* methotrexate, *LEF* leflunomide, *IL* interleukin, *JAK I* Janus kinase inhibitors

Clinical enthesitis was detected in 39.3% of the SLE patients, using Leeds Index; 96.4% of PSA patients; and 17.9% in the healthy controls. When comparing SLE and psoriatic patients, it turned out that PSA patients have a higher frequency of enthesitis clinically using the Leeds Enthesitis Index (96.4%%) compared to SLE (39.3%), with a statistically significant difference, *p* = 0.0001 (Table 2).

**Table 2:** Prevalence LEI of the studied SLE patients, the PSA patients, and the healthy controls

	SLE *n*. 56		PSA *n*. 56		Control *n*. 56		P1	P2	P3
	*n*	%	*n*	%	*n*	%			
LEI	22	39.3	54	96.4	10	17.9	0.0001	0.01	0.0001

*χ*^2^, chi square test

*p* < 0.001, highly significant; *p* < 0.05, significant; *p* > 0.05, non-significant

P1 (compare SLE and PSA), P2 ( compare SLE and healthy control), P3 (compare PSA and healthy controls)

**Fig. 1:**
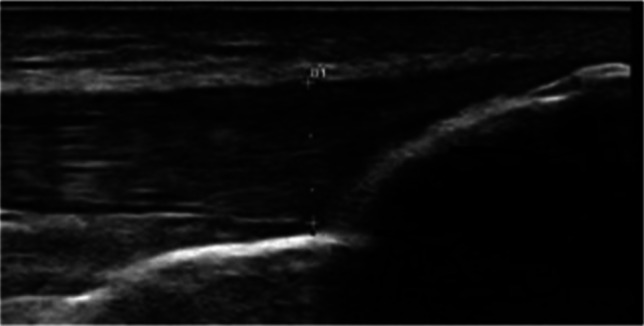
Distal patellar tendon thickening at tibial tuberosity in SLE patients

**Fig. 2:**
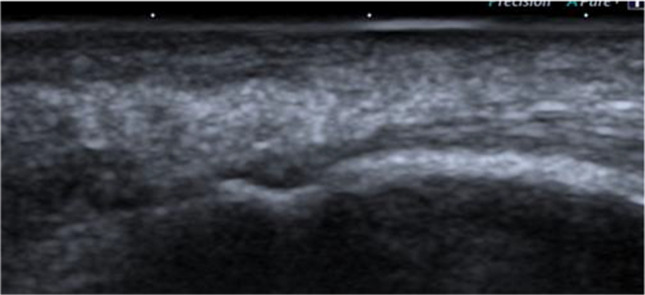
Erosion at triceps tendon insertion in SLE patients

Enthesitis was detected clinically in 39.3 of SLE patients, but the percentage reached 71% by the US, indicating that there was a subclinical enthesitis. The US was highly effective in detecting the enthesitis sites, especially the distal insertion of the patellar tendon at the tibial tuberosity (41% of patients) and the triceps tendon (32.1% of patients) as shown in Table 3 and Figs. 1 and 2.

**Table 3:** Prevalence of the tendon enthesitis via ultrasound examination in the studied groups

Variables	Studied groups						P1	P2	P3
	SLE		Psoriasis		Normal				
	No	%	No	%	No	%			
Triceps tendon	36	32.1	76	67.9	2	1.8	0.0001	0.0001	0.0001
Quadriceps tendon	34	30.4	88	78.6	4	3.6	0.0001	0.0001	0.0001
Inferior pole of patella	28	25.0	52	46.4	0	.0	0.0001	0.0001	0.0001
Tibial tuberosity	46	41.1	74	66.1	4	3.6	0.001	0.0001	0.0001
Achilles tendon	32	28.6	80	71.4	12	10.7	0.0001	0.001	0.0001
Planter fascia	18	16.1	34	30.4	0	.0	0.01	0.0001	0.0001
Total joint affected	80	71.4	112	100.0	22	19.6	0.0001	0.0001	0.0001

*χ*^2^ = chi square test

*p* < 0.001, highly significant; *p* < 0.05, significant; *p* > 0.05, non-significant

P1 (compare SLE and PSA), P2 (compare SLE and healthy control), and P3 (compare PSA and healthy controls)

Ultrasound examination showed that the prevalence of the pathological tendon structure, pathological thickness, infrapatellar bursitis, and retrocalcaneal bursitis was significantly more frequent in PSA patients compared to SLE patients and healthy control *p* < 0.05, while there was no significant difference in the prevalence of pathological calculus and Doppler activity of studied tendons *p* > 0.05, except at the Quadriceps tendon and the Achilles tendon in PSA patients compared to SLE patients *p* < 0.05, as indicated in Table 4.

**Table 4:** Prevalence of the pathological tendons entheses of the studied groups

Variables	Studied groups						P1	P2	P3
	SLE patients		Psoriasis patients		Normal patients				
	No	%	No	%	No	%			
Triceps structure	30	26.8	58	51.8	0	0.0	0.0001	0.0001	0.0001
Triceps thickness	8	7.1	34	30.4	2	1.8	0.0001	0.052	0.0001
Triceps erosion	4	3.6	8	7.1	0	0.0	0.23	0.12	0.007
Triceps calculus	20	17.9	28	25.0	0	0.0	0.19	0.0001	0.0001
Triceps Doppler	2	1.8	8	7.1	0	0.0	0.052	0.49	0.007
Quadriceps structure	24	21.4	80	71.4	0	0.0	0.0001	0.0001	0.0001
Quadriceps thickness	4	3.6	20	17.9	4	3.6	0.001	1	0.0001
Quadriceps erosion	4	3.6	18	16.1	0	0.0	0.002	0.12	0.0001
Quadriceps calculus	16	14.3	28	25.0	0	0.0	0.04	0.0001	0.0001
Quadriceps Doppler	0	0.0	10	8.9	4	3.6	0.001	0.12	0.098
Inferior pole of patella structure	22	19.6	40	35.7	0	0.0	0.007	0.0001	0.0001
Inferior pole of patella thickness	14	12.5	16	14.3	0	0.0	0.69	0.0001	0.0001
Inferior pole of patella erosion	8	7.1	14	12.5	0	0.0	0.18	0.007	0.0001
Inferior pole of patella calculus	10	8.9	12	10.7	0	0.0	0.65	0.001	0.0001
Inferior pole of patella doppler	2	1.8	6	5.4	0	0.0	0.28	0.49	0.029
Tibial tuberosity structure	30	26.8	42	37.5	0	0.0	0.086	0.0001	0.0001
Tibial tuberosity thickness	2	1.8	22	19.6	2	1.8	0.0001	1	0.0001
Tibial tuberosity erosion	20	17.9	34	30.4	0	0.0	0.24	0.0001	0.0001
Tibial tuberosity calculus	12	10.7	18	16.1	0	0.0	0.084	0.004	0.0001
Tibial tuberosity doppler	8	7.1	16	14.3	0	0.0	0.098	0.68	0.018
Tibial tuberosity bursitis	4	3.6	10	8.9	2	1.8	0.029	0.0001	0.0001
Achilles tendon structure	10	8.9	52	46.4	0	0.0	0.0001	0.001	0.0001
Achilles tendon thickness	10	8.9	26	23.2	2	1.8	0.004	0.018	0.0001
Achilles tendon bursitis	12	10.7	28	25.0	6	5.4	0.005	0.14	0.0001
Achilles tendon erosion	8	7.1	26	23.2	0	0.0	0.001	0.007	0.0001
Achilles tendon calculus	2	1.8	18	16.1	0	0.0	0.0001	0.49	0.0001
Achilles tendon doppler	0	0.0	24	21.4	4	3.6	0.0001	0.12	0.0001
Planter aponeurosis structure	6	5.4	28	25.0	0	0.0	0.0001	0.029	0.0001
Planter aponeurosis thickness	12	10.7	12	10.7	0	0.0	1	0.0001	0.0001
Planter aponeurosis erosion	0	0.0	6	5.4	0	0.0	0.029	1	0.029
Planter aponeurosis calculus	0	0.0	4	3.6	0	0.0	0.12	1	0.012
Planter aponeurosis doppler	2	1.8	0	0.0	0	0.0	0.49	0.49	1

*χ*^2^ = chi square test

*p* < 0.001, highly significant; *p* < 0.05, significant; *p* > 0.05, non-significant

P1 (compare SLE and PSA), P2 (compare SLE and healthy control), P3 (compare PSA and healthy controls**)**

Comparing SLE patients with the healthy control group, it turned out that the prevalence of tendon enthesitis was significantly more frequent in SLE patients (39.3%) compared to the healthy control group (17.9%) *p* < 0.05. On the other hand, the US examination detected a higher statistically significant prevalence of the pathological structure; the pathological thickness of the quadriceps tendon, the distal patellar ligament and the Achilles tendon, the inferior pole of the patella; the tibial tuberosity and the Achilles tendon erosion; the pathological calculus of all tendons except Achilles tendon and Planter aponeurosis; and the infrapatellar bursitis. But there was no statistically significant between SLE patients and the healthy control group regarding the prevalence of the pathological Doppler activity of the studied tendons as illustrated in Table 4.

As for the correlation between ultrasonographic entheseal involvement using MASEI and SLE patient characteristics, there was a significant correlation between the MASI and SLEDAI score (*r* = 0.250*, *p* = 0.048), and the total protein in 24 h (*r* = 0.289*, *p* = 0.031). On the other hand, there was an inverse significant correlation between MASI and serum albumin (*r* =  − 0.324*, *p* = 0.015) as indicated in Table 5.

**Table 5:** Correlation between MASEI score and different SLE patient characteristics

Character	MASEI	
	(*r*)	*p*
Age	− 0.173	0.202
Disease duration	− 0.193	0.155
White blood cells	0.189	0.163
Hemoglobin	0.374**	**0.004**
Total platelet count	0.054	0.694
CRP	− 0.076	0.577
ESR	0.05	0.713
Blood urea	0.166	0.222
Serum creatinine	0.195	0.15
Proteinuria	0.289*	**0.031**
Hematuria	− 0.276*	**0.044**
Pyuria	0.021	0.877
Serum albumin	− 0.324*	**0.015**
AST	0.281*	**0.036**
ALT	0.267*	**0.046**
SLEDAI score	0.250*	0.048
Anti-dsDNA	0.245	0.068
C3	0.054	0.695
C4	0.267*	**0.047**

(*r*) correlation coefficient

^**^Correlation is highly significant at the 0.01 level (2-tailed)

^*^Correlation is significant at the 0.05 level (2-tailed)

*C3* complement 3, *C4* complement 4

*p*<0.05 signficant

*p﻿*<0.01 highly significant

Regarding the relation between US pathological findings by MASEI score and the current patients therapies, we found that SLE patients on large corticosteroid doses more than 40 mg per day had a significant lower MASEI scores compared to patients on lower corticosteroids doses, *p* = 0.046. No significant relation was detected with other therapies. In PSA, there is no relation between type of medications and MASEI value, except patients on anti-IL17 that have a significant lower MASE value *p* = 0.014 as indicated in Table 6.

**Table 6:** Relation between medications and MASEI of the studied SLE and PSA patients

SLE	MASEI	*u*	*p*-value	PSA	MASEI	*u*	*p*-value
	Median (range)				Median (range)		
HDQ	6 (0–33) 3 (0–24)	0.676	0.499	NSAIDS: *n* 41 MTX: *n* 48 LEF: *n* 12 Anti-IL 17: *n* 28	19 (3–44) 10 (6–39)	1.095	0.274
AZA	3 (0–24) 5 (0–33)	0.448	0.654		18 (3–44) 19 (3–26)	0.164	0.869
CYC	3 (0–22) 4 (0–33)	0.338	0.698		21 (6–44) 17 (3–44)	1.7	0.089
MMF	5 (0–33) 3 (0–33)	0.232	0.817		17 (3–37) 20 (9–44)	2.46	0.014*
CS							
Large dose	2.5 (0–33)			JAK I: *n* 6	19 (6–44)	0.664	0.507
Low dose	13 (0–33)	1.993	0.046*		18 (3–44)		

*u* = Mann–Whitney *u*; *p* > 0.05, not significant; *p* < 0.05, significant

*AZA* azathioprine, *CYC* cyclophosphamide, *MMF* mycophenolate mofetil, *CS* corticosteroids, *MTX* methotrexate, *LEF* leflunomide, *IL* interleukin, *JAK I* Janus kinase inhibitors

## Discussion

Enthesis is not one of the potential targets of the disease, and the inflammatory process in SLE is traditionally thought to be localized at synovial tissue locations. Ultrasound is a prospective first-line imaging approach for the evaluation of SLE patients with joint complaints [18].

In the present study, subclinical enthesitis was detected in SLE patients by US examination. In agreement with a study [5] including 65 SLE patients, 50 PSA patients, and 50 healthy volunteers, 33.8% and 67.7% of SLE patients showed clinical and ultrasound evidence of enthesitis, whereas at least 94% of PSA patients had one or more abnormalities. Also, in accordance with Elsherbiny et al. [3], 44% of the patients exhibited clinical enthesitis, 58% had clear signs of MSUS enthesitis, and 32% had minor entheseal abnormalities. In this context, a low frequency of enthesitis was detected by Di Matteo et al. [19] who investigated 20 cases and stated that clinical enthesitis symptoms were only seen in one patient, and the MSUS evaluation revealed that four out of twenty SLE patients (20%) had clearly obvious MSUS findings indicative of enthesitis, most frequently at the distal insertion of the patellar tendon. Such data can raise the possibility that enthesis may be a target that has been overlooked in the clinical evaluation of SLE patients.

The amount of structural damage and tendon enthesitis revealed by the US examination was highly frequent in SLE patients than that found in the healthy people and much less than that found in the PSA patients. Similarly to a previous study [5], US enthesitis was more common in SLE patients than in healthy participants: 44 out of 65 SLE patients had enthesitis in comparison with 22 out of 50 healthy subjects (a difference of 44%) (*p* = 0.011) [5].

In the present study, the distal patellar ligament represented the most affected entheseal site in SLE patients. The US pathological findings showed pathological structure (26.8%), bursitis (17.9%), erosion (10.7%), calcification (7.1%), doppler activity (3.6%), and increasing thickness(1.8%). These results are in agreement with Di Matteo et al. [5]. They stated that the distal insertion of the patellar tendon at the tibial tuberosity was the most commonly affected by SLE (36.7%). On the other hand, Elsherbiny et al. [3] reported a distinct enthesitis distribution as the quadriceps tendon insertion at the superior pole of patella represented the most affected entheseal site.

A novel finding in the present study is that the triceps tendon enthesis is also highly more affected (36%) than other entheses. The US pathological findings included pathological structure (26.8%), calcification (17.9%), increasing thickness (7.1%), erosion (3.6%), and Doppler (1.8%).

Di Matteo et al. [5] assumed that enthesitis is thought to be rarely related to US findings of structural damage in SLE patients when compared to PSA or RA but, in the present study, enthesitis in SLE patients was associated with US observations of pathological structure in distal insertion of the patellar tendon and the triceps tendon (26.8%).

When comparing the SLE patients and healthy controls, pathological structure, calcification of triceps, quadriceps tendons, and proximal and distal patellar ligament entheses were significantly more frequent in the studied SLE patients compared to healthy controls. So, in the present study, the US abnormal findings were significantly associated with SLE patients compared to the healthy controls. This result is in agreement with that of Di Matteo et al. who found that US entheseal anomalies were also discovered in healthy people. However, compared to SLE patients, the incidence of US findings indicates that active enthesitis was much lower. In particular, just 2 out of 500 entheses of healthy people had PD signals, in comparison with 49 out of 650 entheses of SLE patients [5].

In the present study, the quadriceps tendon at the superior pole of patella and Achilles tendon represented the most affected entheseal sites in PSA patients. However, Di Matteo et al. [5] and Hussein et al. [13] reported different distributions of enthesitis and stated that distal insertion of the patellar tendon at the tibial tuberosity was the most involved in PSA patients. In the same context, Moshrif et al. [20] and Gutierrez et al. [21] found that Achilles entheses were the most common site for enthesitis via the US examination.

In the present study, PSA patients showed a higher prevalence of US pathological findings in almost all entheses than SLE patients. This result is in agreement with that of Di Matteo et al. [5] since they found that PSA patients showed a higher prevalence of US pathological findings in comparison with SLE patients.

In the present study, there was a significant correlation between the MASI and SLEDAI score and the total protein in 24 h. This result is in agreement with Elsherbiny et al. [3] who found that the scores of Glasgow Ultrasound Enthesitis Scoring System (GUESS) were significantly correlated with age, SLEDAI-2 K, and ESR. In addition, Di Matteo et al. [5] found a positive correlation between the US score of entheseal thickening, hypoechogenicity, Doppler activity, the SLEDAI-2 k, the musculoskeletal-BILAGa, the patient’s age, and the US score of bone erosion and age.

Regarding the current patient’s therapies, SLE patients on large corticosteroid dose had a significant lower MASEI scores and less enthesitis promoting the effect of corticosteroids on the inflammatory process at the sites of enthesitis, while PSA patients on biological therapies have less enthesitis. Up to our knowledge, no previous studies investigated the relationship between the immunosuppressive therapies and enthesitis in SLE; therefore, further studies needed to evaluate this relation and to assess the effectiveness of other therapies as local steroid injections, non-steroidal anti-inflammatory therapies, and conventional and targeted synthetic disease-modifying antirheumatic and biological drugs in the treatment of enthesitis in SLE as the previous therapies were used successfully in psoriatic arthritis patients [22]. The limitation of the study is as follows: the fact that the current study only examined individuals from a single center makes it challenging to apply the findings to the overall lupus population. For a more detailed analysis of the prevalence, characteristics, and clinical importance of entheseal involvement in SLE, the study recommends further investigation.

## Data Availability

All data are available.
